# Influence of Molecular Weight and Degree of Deacetylation of Low Molecular Weight Chitosan on the Bioactivity of Oral Insulin Preparations

**DOI:** 10.3390/md13041710

**Published:** 2015-03-27

**Authors:** Nidal A. Qinna, Qutuba G. Karwi, Nawzat Al-Jbour, Mayyas A. Al-Remawi, Tawfiq M. Alhussainy, Khaldoun A. Al-So’ud, Mahmoud M. H. Al Omari, Adnan A. Badwan

**Affiliations:** 1 Department of Pharmacology and Biomedical Sciences, Faculty of Pharmacy and Medical Sciences, University of Petra, P.O. Box 96134, Amman 11196, Jordan; E-Mails: nqinna@uop.edu.jo (N.A.Q.); KarwiQG@cardiff.ac.uk (Q.G.K.); tawfikh@uop.edu.jo (T.M.A.); 2 The Jordanian Pharmaceutical Manufacturing Company (PLC), Research and Innovation Centre, P.O. Box 94, Naor 11710, Jordan; E-Mails: nawzat@jpm.com.jo (N.A.-J.); momari@jpm.com.jo (M.M.H.A.O.); 3 College of Pharmacy, Taif University, Taif 5700, Saudi Arabia; E-Mail: mayyas@tu.edu.sa; 4 Department of Chemistry, Faculty of Science, Al al-Bayt University, P.O. Box 130040, Mafraq 25113, Jordan; E-Mail: khaldoun@aabu.edu.jo

**Keywords:** insulin, oral delivery, oligochitosan, low molecular weight chitosan, nanoparticles, spectroscopy, glucose, diabetic rats

## Abstract

The objective of the present study was to prepare and characterize low molecular weight chitosan (LMWC) with different molecular weight and degrees of deacetylation (DDA) and to optimize their use in oral insulin nano delivery systems. Water in oil nanosized systems containing LMWC-insulin polyelectrolyte complexes were constructed and their ability to reduce blood glucose was assessed *in vivo* on diabetic rats. Upon acid depolymerization and testing by viscosity method, three molecular weights of LMWC namely, 1.3, 13 and 18 kDa were obtained. As for the DDA, three LMWCs of 55%, 80% and 100% DDA were prepared and characterized by spectroscopic methods for each molecular weight. The obtained LMWCs showed different morphological and *in silico* patterns. Following complexation of LMWCs with insulin, different aggregation sizes were obtained. Moreover, the *in vivo* tested formulations showed different activities of blood glucose reduction. The highest glucose reduction was achieved with 1.3 kDa LMWC of 55% DDA. The current study emphasizes the importance of optimizing the molecular weight along with the DDA of the incorporated LMWC in oral insulin delivery preparations in order to ensure the highest performance of such delivery systems.

## 1. Introduction

Different formulation strategies have been investigated to overcome the gastrointestinal tract (GIT) barriers for the delivery of proteins via the oral route. These different delivery systems included microemulsions, nanoparticles and coated liposomes [1]. By using these delivery forms, it has been found that nanosized systems may offer a reasonable solution for oral protein administration [2].

The nanosized particles can be obtained either by ultra homogenization, which may expose protein to mechanical stress leading to instability, or by facilitating the formation of nano vesicles using self-emulsifying surface active agents. Among these surface active agents are caprylocaproyl macrogolglycerides and polyglyceryl-6 dioleate. These surfactants are self-emulsifying in aqueous media and form reversible micelles when dispersed in oils. In previous work however, we have confirmed that the addition of low molecular weight chitosan (LMWC) of different molecular weights to a dispersion of the two surfactants mixed in 1:1 ratio in oleic acid had resulted in the formation of reversed micelles that were shrinked to nano size vesicles [3].

Indeed, a wide range of biodegradable and conventional polymers has been investigated in forming nanosized particles. Among these polymers is chitosan, which is well established as a safe food and drug additive [4,5]. Chitosan chemically is a linear co-polymer consisting of β (1-4)-linked 2-amino-2-deoxy-d-glucose (d-glucosamine) and 2-acetamido-2-deoxy-d-glucose (*N*-acetyl-d-glucosamine) units. Chitosan is mainly identified and characterized by three parameters; molecular weight, DDA, and polydispersity. It is unfortunate that in most protein delivery studies high molecular weight chitosans (HMWC)s were used. In many cases, the molecular weights of the used chitosans were not predetermined. Furthermore, using the insoluble HMWCs limited their application in liquid protein delivery dosage forms. This makes using LMWCs in such preparations more flexible.

LMWCs can be mainly prepared from the HMWCs using the acidic depolymerization, oxidative, enzymatic, or ultrasonic degradation [6]. Naturally, the process of deacetylation of chitosan involves the removal of acetyl groups from the molecular chain of the polymer, leaving behind an exposed amino group (–NH_2_) [7,8]. This positively charged group facilitates the chitosan involvement in forming more electrostatic complexes with the negatively charged molecules such as insulin amino acids. Consequently, these polyelectrolyte complexes (PECs) could be loaded with proteins resulting in micro or nano-carrier systems [9,10]. On the other hand, reducing the DDA of chitosan can be achieved by the addition of acetyl groups to the chains of the polymer by reacting chitosan solution with acetic anhydride [11]. These added groups could also influence PEC formation, and therefore, must be investigated.

Previously, a delivery system based on preparing a PEC between LMWC (13 kDa, 80 DDA%) and insulin was reported [12]. A mixture of caprylocaproyl macrogol-8-glycerides, polyglyceryl-6 dioleate and oleic acid was used to prepare the reverse micelles. This delivery system showed a reduction in glucose level in diabetic rats [13]. Furthermore, this delivery system was tested in more than 50 healthy volunteers using euglycemic technique and results showed a successful reduction in their blood glucose levels [14].

Although the used chitosans in these previous studies were purified and characterized, the impact of selecting different molecular weights and DDAs of chitosan in such nanosized preparations was not investigated. Consequently, the main aim of this study was to explore the influence of LMWCs of different DDAs on the formation of nanosized vesicles for protein delivery and its impact on insulin’s *in vivo* absorption.

## 2. Results and Discussion

Many approaches have been employed to deliver proteins orally including the use of specific excipients, such as absorption enhancers, enzyme inhibitors and mucoadhesive polymers. Despite these various attempts, however, no clinically useful oral formulations have been developed until now [15].

Chitosan is a biocompatible and biodegradable polymer that has permeability enhancing and mucoadhesive properties [16,17]. These properties made the chitosan be a polymer of choice for protein delivery. While chitosan shows important functional properties, nevertheless, the high molecular weight, high viscosity and insolubility at physiological pH of chitosan restrict its use *in vivo* [18]. Indeed, the intestinal absorption of LMWC can be significantly better than that of HMWC [19]. Therefore, nanocarriers research is currently directed towards using LMWCs in drug delivery [20].

### 2.1. Preparation and Characterization of LMWCs with Different DDA

LMWCs with different molecular weights (1.3, 13, and 20 kDa) and DDA (55%, 80%, and 100%) were prepared from HMWC (250 kDa, 93% DDA). Initially, fully acetylated LMWCs were prepared by the acid depolymerization method. Table 1 shows their corresponding molecular weights as a result of changing the reaction time from 2, 3.2, to 24 h. The molecular weights, as determined by viscosity measurements, were 1.25, 12.75 and 18.2 kDa. For convenience, these molecular weights were named 1.3, 13 and 18 kDa.

To prepare LMWCs with different DDA (55%, 80% and 100%), the obtained fully deacetylated LMWCs (1.3, 13 and 18 kDa) were reacted with acetic anhydride using different molar ratio (Table 1). DDAs (55%, 80% and 100%) of each LMWC were determined by UV/visible spectrophotometry and verified using the ^1^H-NMR spectrometry. The DDA values obtained by both methods were comparable (Table 1).

**Table 1:** Experimental data of molecular weight (MW) and degree of deacetylation (DDA) determinations for low molecular weight chitosans (LMWCs).

Molecular Weight											
LMWC MW (kDa)	1.3					13			18		
Depolymerization time (h)	24					3.2			2.0		
Experimental MW (kDa)	1.25					12.75			18.20		
**Degree of Deacetylation (DDA)**											
DDA %		55			80				100		
Molar ratio of chitosan:Ac_2_O		1:0.60			1:0.15				1:0		
LMWC MW (kDa)		1.3	13	18	1.3		13	18	1.3	13	18
Experimental DDA%											
By UV/visible		54.9	54.8	55.1	79.8		80.3	80.2	99.6	99.4	100.3
By ^1^H-NMR		55.0	55.4	56.6	79.6		79.1	80.6	100.0	100.0	100.0

**Figure 1:**
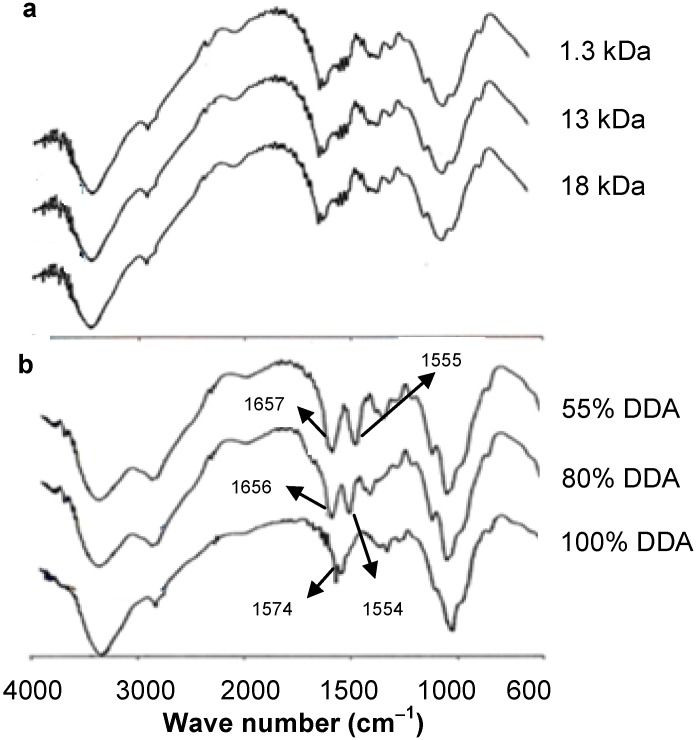
FT-IR spectra over the frequency range 4000–400 cm^−1^: (**a**) different molecular weight chitosans (LMWCs) of fully deacetylated and (**b**) 13 kDa LMWC of different degree of deacetylation (DDA).

The FT-IR spectra of chitosan, in general, show a broad absorption band in the range 3000–3500 cm^−1^ attributed to O–H and N–H stretching vibrations, while the peaks around 2885, 1650, 1589, 1326 and 1080 cm^−1^ are due to the stretching vibrations of aliphatic C–H, C=O stretching in secondary amide (Amide I), free amino –NH (Amide II) and C–N–stretching in secondary amide (Amide III) and C–O–C, bonds, respectively [21]. In the present work, however, the formation of fully deacetylated LMWC was confirmed by FT-IR with the absence of Amide I peak at 1650 cm^−1^ (Figure 1a), while the peak corresponding to free amino band (Amide II) appeared at 1574 cm^−1^. This result is in agreement with that reported by Heux *et al.* [22]. It is worth mentioning that neither the acid hydrolysis nor the acetylation reactions altered the skeleton structures of the obtained LMWCs (Figure 1a,b).

**Figure 2:**
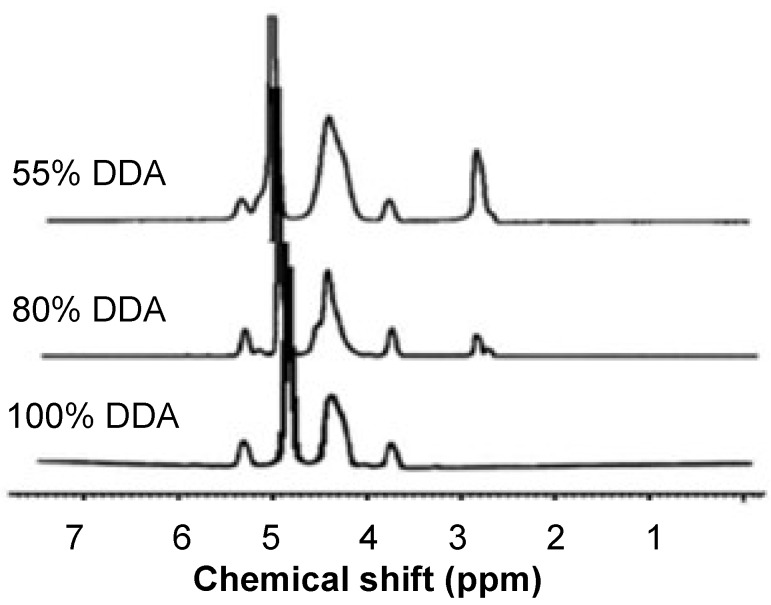
^1^H-NMR spectra for 13k Da low molecular weight chitosan (LMWC) of different degree of deacetylation (DDA).

The acetylation of LMWC was monitored by ^1^H-NMR spectroscopy, as shown in Figure 2. It was observed that the integral of the peak of the three protons of acetyl group (H-Ac) at 2.8 ppm increases by decreasing the DDA values (Figure 2).

### 2.2. Surface Morphology

**Figure 3:**
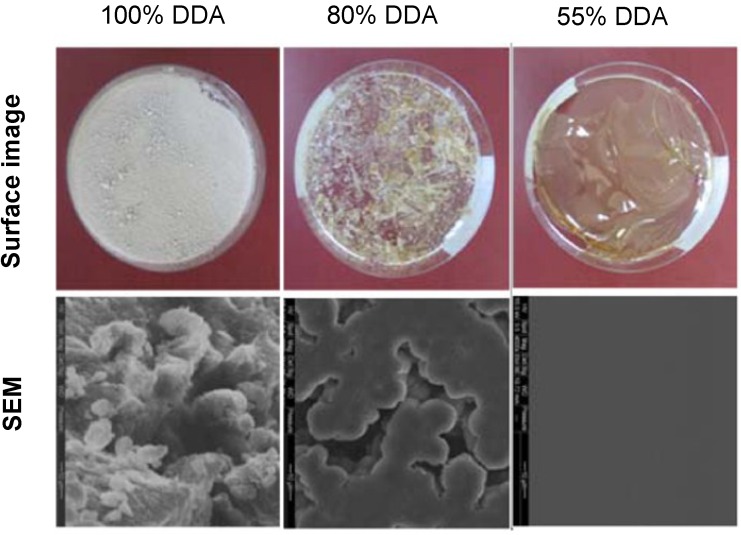
Surface images and scanning electron microscope captures of dry films of 13 kDa low molecular weight chitosan (LMWC) of different degree of deacetylation (DDA), 4000× magnification.

The prepared LMWCs showed variations in their surface morphology as shown by the photos of their thin films (Figure 3). The crystallization and the trend of film formation were increased as long as the DDA decreased. At the lowest DDA (55%), chitosan was arranged in the form of fibers that differ in their thickness and flexibility. Furthermore, the higher molecular weights had thicker and more rigid fibers compared with the lower molecular weights that had thin and fragile fibers.

The Scanning Electron Microscope images of different LMWCs (Figure 3) showed that all fully deacetylated LMWCs had rough and irregular surfaces. At the DDA around 80%, the *N*-substituted polymer chains were in good morphological sphericity and aggregated to each other like rosaries. At the DDA decreased (55%), the surface shape of the LMWCs was regular, smooth, stretched and looked like a continuous flat section.

### 2.3. In Silico Characterization

**Figure 4:**
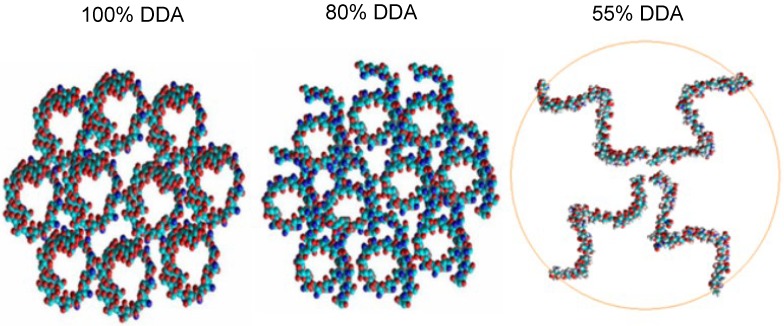
*In silico* arrangements of low molecular weight chitosans (LMWCs) each constructed from 50 units of d-glucosamine (10 kDa) having different degree of deacetylation (DDA).

The *in silico* simulations (Figure 4) showed that the best orientation of the deacetylated LMWC occurred when the constructed chitosan oligomers had been arranged as spheres bound together. When the DDA was decreased to 80%, the mode of binding between the polymer chains became helical in shape. However, chitosan oligomers with the least DDA (55%) arranged themselves in small aggregates each containing 4–5 chains constructed from glucosamine units. The best orientation of LMWC polymer with DDA of 55% incorporated only four chains while the number of chains reached up to 10 for higher DDA (>80%). It was also noted that as the DDA decreases, the intermolecular forces between the polymer chains also decrease and produce more flexible chains. Therefore, it can be predicted while constructing the PEC that the number of binding sites between insulin and LMWC would increase as the DDA decreases.

### 2.4. Oral Insulin Formulation and Aggregation Size of the Reverse Micelle

**Figure 5:**
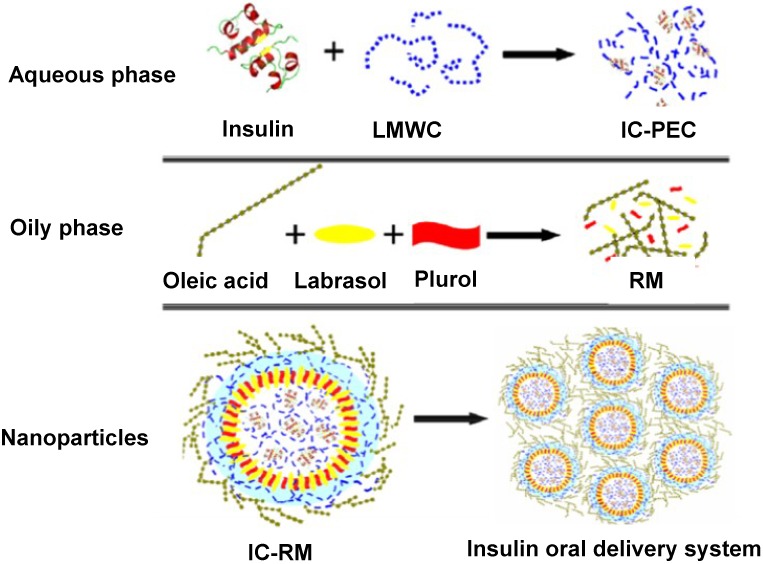
Illustration of the essential components of the prepared insulin-chitosan polyelectrolyte complex (IC-PEC) and its revised micelles (IC-RM). LMWC represents low molecular weight chitosan.

The tested nanoparticle system can be described as a w/o microemulsion constructed by mixing LMWC with insulin to form a PEC (aqueous phase) which was then solubilized in a mixture made from Labrasol^®^ and Plurol Oleique^®^ dissolved in oleic acid (Figure 5). The surfactant-cosurfactant system namely, Labrasol and Plurol, was prepared at 1:1 mass ratio as previously reported [3] despite the fact that many researchers often use the 4:1 mixing ratio as delivery vehicles [23,24].

Chitosan, as a polyelectrolyte, can form electrostatic complexes under acidic conditions. Two different types of complexes are considered, electrostatic complexes with an oppositely charged surfactant and PECs [25]. In fact, protein-PEC are not new and have been used extensively in biology over many years for protein purification, immobilization and stabilization of enzymes [26].

**Figure 6:**
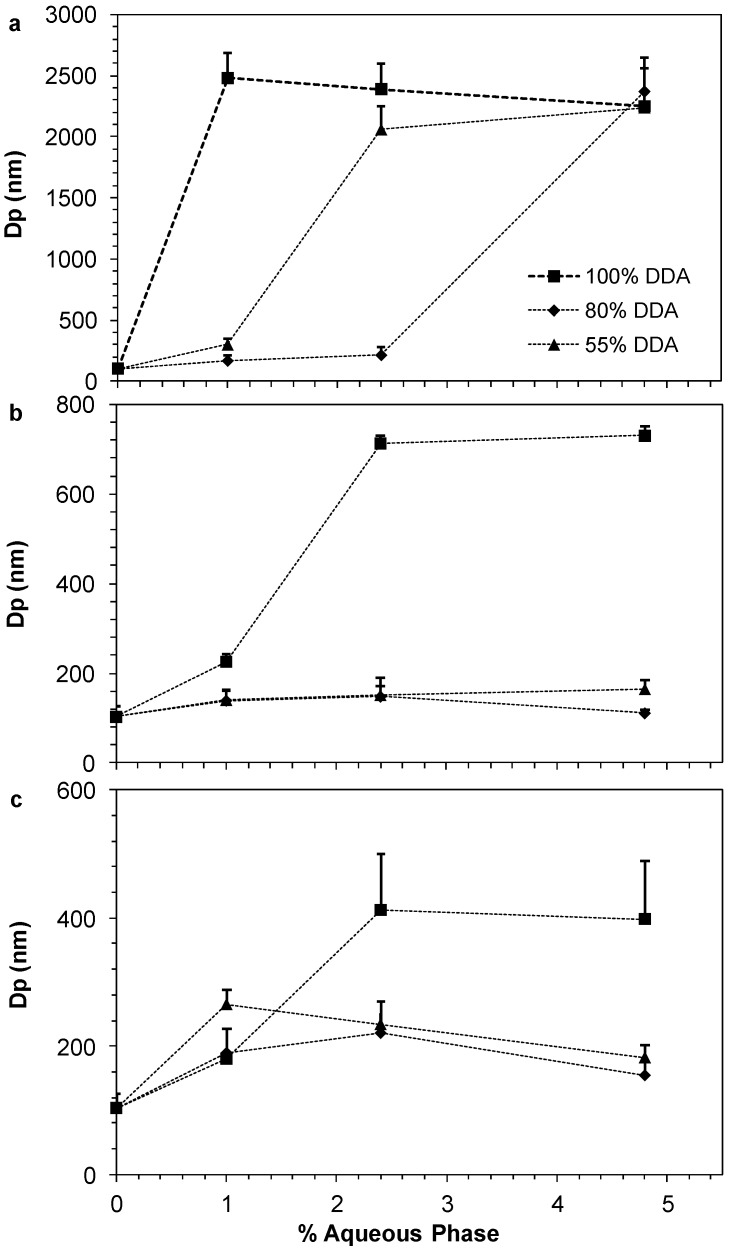
Effect of changing the percentage of aqueous phase on the aggregation size of oral insulin preparations loaded with different degree of deacetylation (DDA) of low molecular weight chitosan (LMWC): (**a**) 1.3 kDa; (**b**) 13 kDa; and (**c**) 18 kDa. Data are presented as mean ± SD (*n* = 8).

The relationship between the particle diameter and the amount of the aqueous phase loaded in the reverse micelle system was established for each LMWC. Figure 6 clearly shows that loading of insulin-chitosan PEC in the oleic acid and surfactant/cosurfatant mixture increased the diameter of the nanoparticles. When using 1.3 KDa LMWC with different DDA in the preparations (Figure 6a), large particle sizes of more than 2000 nm were obtained. However, increasing the molecular weight of the LMWC significantly reduced the particle size of the formed nanoparticles (Figure 6c). For all LMWCs, it can also be noted that the fully deacetylated LMWCs failed to maintain small particle sizes when the amount of the aqueous phase was increased. For example, the particle size of the reverse micelle (RM), prepared by 13 kDa LMWC of 80% DDA, decreased to be in the vicinity of 150 nm, while it reached around 700 nm when the fully deacetylated LMWC (100% DDA) was used in the RM preparation.

Therefore, the DDA of the incorporated LMWC in oral insulin preparations can have a large influence on the formation and properties of stable solubilized reverse micellar systems as chitosan was found to possess certain surface activity in the presence of oleic acid as previously reported by Assaf *et al.* [3].

### 2.5. In Vivo Pharmacological Activity of the Nano Dispersion System

**Figure 7:**
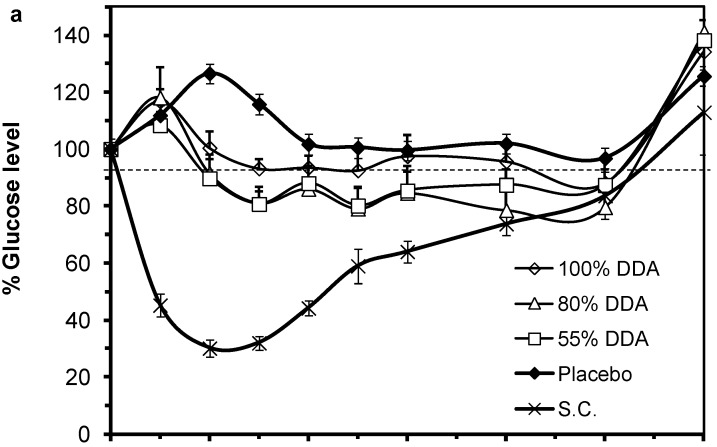

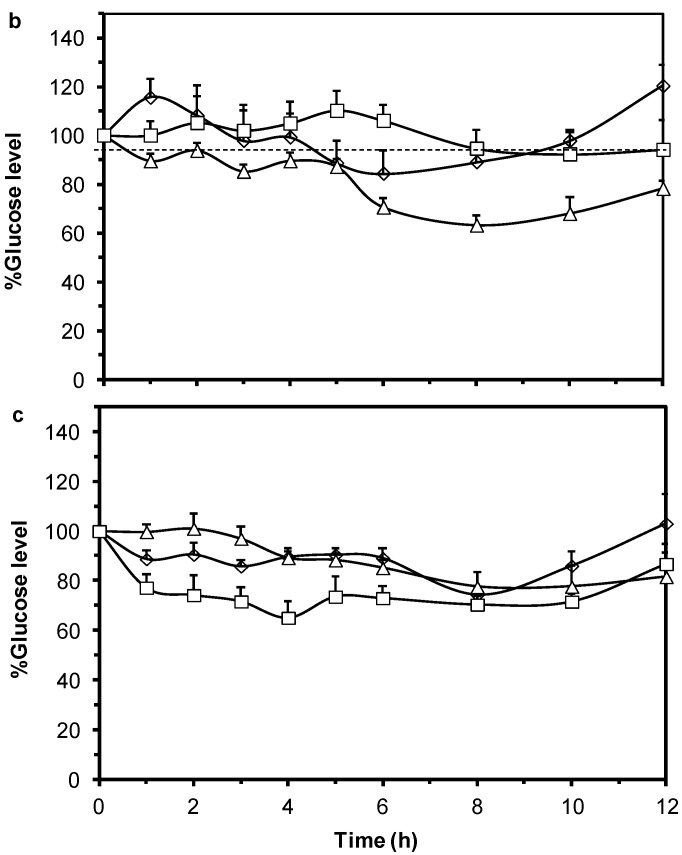
Percentage change of blood glucose levels of diabetic rats administered oral insulin (50 IU/kg) preparations loaded with different degree of deacetylation (DDA) of low molecular chitosans (LMWCs): (**a**) 1.3 kDa along with a S.C. insulin (1 IU/kg) and a placebo group; (**b**) 13 kDa and (**c**) 18 kDa. Data points are represented as mean ± SEM (*n* = 10). The chitosan concentration was fixed at 4.8 μmol/mL in all oral insulin preparations.

The effect of different preparations of LMWCs with different DDA on blood glucose levels of streptozotocin induced diabetic Sprague Dawley rats are presented in Figure 7. Rats subcutaneously injected with insulin (1 IU/kg) showed an intense response of glucose reduction while the placebo preparations did not reduce blood glucose levels. Weak response to insulin was obtained when using the 1.3 kDa LMWC in IC-RM preparations. The glucose reduction was not significant in the 100% DDA preparations while better efficacy patterns, though still weak, were obtained with 80% DDA and 55% DDA (Figure 7a). Such weak response might be attributed to the larger particle size obtained when using the 1.3 kDa LMWC in the preparations as described in Figure 6a.

As for using the 13 kDa LMWC in the preparations (Figure 7b), the best reduction of glucose level was achieved with the 80% DDA where a maximum and significant decrease of 63% ± 3.9% was obtained after 8 h post administration (*p* < 0.05). In Figure 7c, the preparation containing 18 kDa LMWC/55% DDA showed the best efficacy among the other formulas where the maximum reduction in blood glucose level was achieved after 4 h of the oral dose administration and reached 65% ± 4.2%. This reduction was considered efficient as no statistical significant difference was seen when compared to the subcutaneous (S.C.) group (*p* = 0.142). Moreover, in the case of 13 and 18 kDa LMWCs, two way analysis of variance for repeated measures revealed that glucose levels were significantly changed when different DDA were used (*p* < 0.01). Such effect of DDA was not statistically significant in the case of 1.3 kDa (*p* = 0.289).

**Figure 8:**
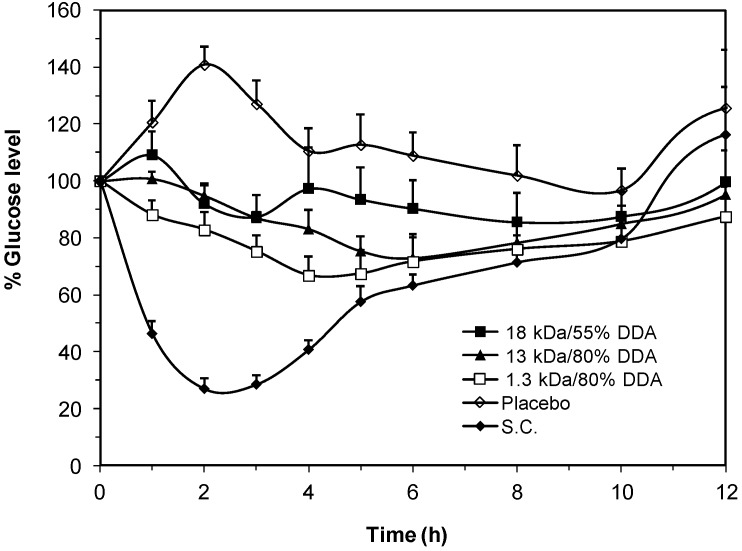
Percentage change of blood glucose levels of diabetic rats administered oral insulin (50 IU/kg) preparations loaded with LMWCs of 1.3 kDa/80%DDA; 13 kDa/80%DDA and 18 kDa/55%DDA and subcontenous (S.C.) insulin (1 IU/kg) compared to a placebo group. The chitosan concentration was fixed at 62.4 mg/mL in all oral insulin preparations. Data points are represented as mean ± SEM (*n* = 10).

The above tested oral insulin dispersions (Figure 7) were prepared by including a fixed molar concentration (4.8 μmol/mL) of each LMWC in the preparation. However, in order to exclude the variability in the amount of LMWC incorporated in the preparation of IC-PECs in, the three formulae that showed the best extent of insulin release in the above studies were formulated to contain equal and sufficient amounts of LMWC (62.5 mg/mL) that is enough to form PEC with the whole amount of insulin in the formulae. *In vivo* results for these preparations showed that all preparations reduced significantly blood glucose levels compared to the placebo group. The formula of 1.3 kDa LMWC/80% DDA had a unique linear but rather none statistically significant pattern of glucose reduction as compared with other formulas (Figure 8). The maximum limit of blood glucose reduction (66% ± 5.9%) was obtained after 4 h of oral administration of the formula. Moreover, when comparing the formula of 13 kDa LMWC/80% DDA with the 18 kDa LMWC/55% DDA formula, Tukey’s post-test revealed a significant difference in the activity between the 4th and 6th hour sampling intervals (*p* < 0.05). Therefore, other than the molecular weight and the DDA of chitosan, it seems also necessary to optimize the amount of chitosan that should be included in the formation of insulin nanoparticles. Such amount should be sufficient to interact with its counterpart electrolye during PEC formation and sufficient for protecting the outer layer of the formed aqueous nano sized droplets.

## 3. Experimental Section

### 3.1. Materials

Recombinant human (rh-)insulin (99.4%) was obtained from Biocon Limited (Electronic City, Bangalore, India). Calbiochem^®^ Streptozotocin (STZ) and oleic acid vegetable were obtained from Merck KGaA (Darmstadt, Germany). Labrasol^®^ (PEG-8 caprylic/capric glycerides, HLB 14) and Plurol^®^ Oleique CG (Polyglycerol-6-dioleate, HLB 6) were obtained from Gattefosse (Saint-Priest, Lyon, France). Chitosan (Molecular weight ~250 kDa and DDA ~93%) was obtained from Xiamen Xing (Shanghi, China). Purified water was obtained from the Jordanian Pharmaceutical Manufacturing (JPM) company (Naor, Jordan), which was double distilled (Conductivity < 2 μs/cm) prior its use. All other chemicals were of analytical grade.

### 3.2. Preparation of Fully Deacetylated LMWCs

Different LMWCs (1.3, 13, and 18 kDa) were prepared by acid depolymerization as reported earlier [5,27]. Chitosan (250 kDa, 93% DDA) solutions (1% in 2 M HCl) were refluxed for 2, 3.5 and 24 h to get LMWCs of 18, 13 and 1.3 kDa, respectively. At the end of the reaction, chitosan solution was added to 96% ethanol (1:2 *v*/*v* aqueous to ethanol ratio). After cooling, the precipitate was filtered and washed several times with ethanol, followed by centrifugation at 4000 rpm for 5 min (accuSpin™ 3 centrifuge, Fisher Scientific, Schwerte, Germany) to get neutral pH (6.8–7) filtrate. The obtained LMWCs as HCl salt were then freeze dried for 24 h (Hetopower dry PL 9000 freeze drier, Thermo Fisher Scientific, Inc., Waltham, MA, USA).

### 3.3. Preparation of LMWCs with Different DDAs

LMWCs with DDA of 55% and 80% were prepared by reacting fully deacetylated LMWC with acetic anhydride at molar ratios of 1:0.15 and 1:0.6, respectively. LMWC solution (1% in water) was initially prepared and the pH was adjusted to 6.5 by drop wise addition of 6 M NaOH. Acetic anhydride was then added (1 and 2 portions for 55% and 80%, respectively) with magnetic stirring (500 rpm) at room temperature for 10 min [28]. The pH of the solution was maintained at 6.25 after the addition of each portion of acetic anhydride. The chitosan solution was then dialyzed against 4 L of distilled water under gentle stirring at room temperature for 24 h using dialysis tubes having 1000 Da molecular weight cut-off used for 1.3 KDa LMWC and 12,000–14,000 Da cut-off tubes used to dialyze 13 and 18 kDa LMWCs (Medicell International Ltd., London, UK). Finally, the dialyzed chitosan solution was poured in Petri dishes and dried overnight in an oven at 40 °C, then transferred to an amber-airtight glass bottle and stored at room temperature until used.

### 3.4. Determination of the Molecular Weight of LMWCs

The average molecular weight of the prepared LMWCs was calculated from the measured intrinsic viscosity (Vibro-viscometer SV-10, A&D Company, Tokyo, Japan) using a previously reported method [13].

### 3.5. Determination of the DDA

The DDA of the prepared LMWCs was determined by a UV-spectrophotometric method (Beckman Coulter spectrophotometer, DU 640i, Brea, CA, USA) adapted in the European Pharmacopeia (2012). The first derivative at 202 nm was measured for each LMWC dissolved in water (50 μg/mL). The pH of the solution was then measured and the DDA was determined using *N*-acetylglucosamine as a reference to construct a calibration curve with concentrations of 1.0, 5.0, 15.0 and 35.0 μg/mL in water. ^1^H-NMR (Bruker Avance Ultra Shield 300 MHz Spectrometer, Billerica, MA, USA) was used to confirm the obtained results as reported earlier [29]. At least 64 scans were acquired for each LMWC sample (30 mg/mL in D_2_O). The DDA was calculated using integrals of the peak of proton H1 of deacetylated monomer (H1-D) at 5.2 ppm and the peak of the three protons of the acetyl group (H-Ac) at 2.8 ppm.

### 3.6. FT-IR Spectroscopy

The prepared LMWCs were characterized by FT-IR (Thermo scientific Nicolet Avatar 360 FT-IR ESP Spectrometer, Madison, WI, USA) using KBr pellet method.

### 3.7. Surface Morphology

The films of LMWCs, obtained after freeze drying on glass Petri dishes for 24 h, were visually examined and photographed using a digital camera (Sony Cybershot, Tokyo, Japan). Furthermore, the morphological differences of LMWCs were observed using Scanning Electron Microscopy (FEI Quanta 200, Hillsboro, OR, USA) equipped with EDAX for X-ray microanalysis. The photomicrographs were taken at the same magnifications to facilitate comparison between them.

### 3.8. In Silico Molecular Mechanic Modeling

LMWC was built up from 50 units of d-glucosamine (10 kDa) using Hyperchem software (release 8.06) (Hypercube Inc., Gainesville, FL, USA). Three different kinds of LMWCs were built up with different DDA namely 100, 80 and 55%. Several trials were carried out to find the most suitable interaction between the constructed LMWCs.

### 3.9. Formulation of the Oral Insulin Nanoparticle Dispersion System

The oral insulin reverse micelles were prepared according to a method described by Elsayed *et al.* [13]. For the PEC preparation, a predetermined amount of each of the LMWCs was dissolved in 2 mL water. The pH of each solution was adjusted to 6.25 using 2 M NaOH, and the final volume was completed to 4 mL with water reaching a final LMWC concentration of 4.8 μmol/mL. Insulin solution was prepared by dissolving 100 mg of rh-insulin in 1 mL of 0.1 N HCl by gentle shaking. The pH was adjusted to about 8.5 by 0.1 M NaOH, followed by completing the final volume to 4 mL with water reaching a final insulin concentration of 2.15 μmol/mL. The IC-PEC (aqueous phase) was prepared by mixing the insulin and the prepared PEC solutions at room temperature by gentle stirring. The RM system was prepared by mixing Labrasol^®^–Plurol Oleique^®^ (1:1 molar ratio) and oleic acid in a ratio of 20:80 (*w*/*w*) by mixing using a vortex.

For particle size investigations, different amounts of the aqueous phase (0.02, 0.05, 0.1, 0.15 g) were placed in tubes containing 2 g of the RM mixture in order to obtain the maximum loading capacity of the two phases. Subsequently, the tubes were vortexed for 30 s and held on a rack for 15 min at room temperature to equilibrate before measuring their aggregation sizes.

### 3.10. Aggregation Size Determinations of the Nanoparticle Dispersion System

The size of each prepared dispersion system was assessed by dynamic light scattering using a Malvern Zetasizer Nano-ZS series (Malvern Instruments, Worcestershire, UK) at 25 °C with a detection angle of 173. Eight measurements were conducted as the instrument automatically determines the number of runs in each measurement. The instrument built-in software also calculated the average and standard deviations of aggregation size measurements for each percent of loading.

### 3.11. In vivo Studies on Streptozotocine (STZ) Diabetic Rats

#### 3.11.1. Animal Handling

Adult male Sprague Dawley rats with an average weight of 220 ± 20 g were purchased from Yarmouk University (Irbid, Jordan) and accommodated at Petra University Animal House Unit under standard temperature, humidity and photoperiod light cycles. All rats were acclimatized for ten days before experimenting day and received standard chow and tap water. All experiments were carried out in accordance with the guidelines of the Federation of European Laboratory Animal Sciences Association (FELASA). The study protocol was approved (Approval No. PHARM3/11/2012, 3 November 2012) by the Ethical Committee of the Higher Research Council at the Faculty of Pharmacy, University of Petra (Amman, Jordan).

#### 3.11.2. Induction of Diabetes using Streptozotocin

Diabetes was induced in male S.D. rats by intraperitoneal injection of two doses of 80 mg/kg streptozotocin (STZ) over two days. STZ was freshly dissolved in 0.1 M citrate buffer (pH 4.5). Blood glucose level was monitored by measuring glucose concentration in blood samples obtained from the tail vein of rats using a blood glucose meter (GluChec^®^, KMH Co. Ltd., Gyeonggi-do, Koria). Only rats with a basal blood glucose level above 200 mg/dL were considered diabetic.

#### 3.11.3. Pharmacological Activity Evaluations of the Insulin-Loaded Dispersions

Overnight fasted STZ diabetic rats were divided randomly into five groups where each group contained ten diabetic rats. The first three groups received 50 IU/kg oral insulin preparations using oral gavage needle. The fourth group was injected subcutaneously with 1 IU/kg rh insulin solution while another group served as a control and given an oral placebo preparation (nanoparticles dispersion without insulin) through oral gavage. The blood sampling for blood glucose concentration measurement proceeded along the experiment at specific time intervals (0, 1, 2, 3, 4, 5, 6, 8, 10 and 12 h). The zero-time intervals represented the baseline glucose level for the subsequent time intervals.

### 3.12. Statistical Evaluation

One-way analysis of variance (ANOVA) followed by the Tukey’s post-test was used to analyze the differences between groups and differences between time intervals amongst groups using SPSS 17 statistical package, USA. Two-way analysis of variance test for repeated measures was also used to analyze changes in glucose levels. A probability value <0.05 was considered the minimum level of statistical significance. The average aggregation sizes (in nM) are expressed as means ± SD (standard deviation) while blood glucose levels are presented as a percentage of control and expressed as means ± SEM (standard error of means).

## 4. Conclusions

The current investigation showed that the prepared different LMWC had different physicochemical characteristics. Such characteristics are known to influence the stability and strength of the formed polyelectrolyte complexes when react with oppositely charged molecules such as proteins [30]. However, changing the molecular weight or the DDA of the utilized LMWC can also influence the characteristics of the formed nanoparticle system. Some acetylation degree was seen essential to achieve better reduction in the particle size of the formed nanoparticles. On the other hand, when the 1.3 kDa LMWC was used in the preparations, the activity of insulin was only attractive when the amount of chitosan was increased. Such an increase in the incorporated amount of LMWC in the preparation would allow a sufficient level of interaction with insulin while protecting the outer layer of the formed nanoparticle. Therefore, the current study emphasizes the importance of optimizing the molecular weight along with the DDA and concentration of the incorporated LMWC in oral insulin delivery preparations in order to ensure the highest performance such systems.
